# The Role of Cortisol Secretion in Pheochromocytomas and Paragangliomas: Clinical and Perioperative Implications

**DOI:** 10.1210/clinem/dgaf361

**Published:** 2025-06-19

**Authors:** Karolina Zawadzka, Jan Calissendorff, Ewelina Rzepka, Michał Pędziwiatr, Alicja Hubalewska-Dydejczyk, Henrik Falhammar

**Affiliations:** Doctoral School of Medical and Health Sciences, Jagiellonian University Medical College, 31-008 Krakow, Poland; Department of Molecular Medicine and Surgery, Karolinska Institute, 171 76 Stockholm, Sweden; Department of Endocrinology, Karolinska University Hospital, 171 76 Stockholm, Sweden; Chair and Department of Endocrinology, Jagiellonian University Medical College, 30-688 Krakow, Poland; 2nd Department of General Surgery, Jagiellonian University Medical College, 30-688 Krakow, Poland; Chair and Department of Endocrinology, Jagiellonian University Medical College, 30-688 Krakow, Poland; Department of Molecular Medicine and Surgery, Karolinska Institute, 171 76 Stockholm, Sweden; Department of Endocrinology, Karolinska University Hospital, 171 76 Stockholm, Sweden

**Keywords:** paraganglioma, mild autonomous cortisol secretion, cardiovascular disease, diabetes, perioperative complications, metanephrine concentrations

## Abstract

**Background:**

Pheochromocytomas and paragangliomas (PPGLs) are tumors marked by excessive catecholamine secretion. Patients with pheochromocytomas may have elevated plasma glucocorticosteroid concentrations. This study aimed to evaluate the prevalence, clinical implications, and perioperative outcomes of autonomous cortisol secretion in patients with PPGLs.

**Design:**

This was a retrospective cohort study conducted across 2 tertiary endocrinology centers, including patients with PPGLs who underwent adrenalectomy or extra-adrenal surgery for paragangliomas.

**Methods:**

Patients were divided based on the 1-mg dexamethasone suppression test (DST) results into suppressive and nonsuppressive groups (above or below 1.8 µg/dL [50 nmol/L]). Data on clinical characteristics, biochemical markers, tumor features, perioperative outcomes, and follow-up were analyzed.

**Results:**

Among 106 patients, 24.5% exhibited nonsuppressive cortisol concentrations post-DST. These patients were older (median age: 66 vs 56 years, *P* < .001), predominantly female (84.6% vs 48.8%, *P* = .001), and presented with larger tumors (5.2 vs 4.0 cm, *P* < .05). Diabetes was more common in the nonsuppressive group both before adrenalectomy/surgery (50.0% vs 26.8%, *P* < .05) and after (33.3% vs 12.7%, *P* < .05). The nonsuppressive group had higher urinary and plasma metanephrine concentrations, lower DHEAS concentrations, and more cardiovascular diseases. Perioperative complications, including blood loss, conversion to open surgery, and prolonged hospital stays, were more frequent in the nonsuppressive group (*P* < .05).

**Conclusion:**

One-quarter of patients with PPGLs exhibit autonomous cortisol secretion, associated with larger tumors, higher diabetes prevalence, and increased perioperative risks. Routine DST screening may improve preoperative management and offer insights into the impact of cortisol on PPGLs outcomes.

Pheochromocytomas and paragangliomas (PPGLs) are primarily characterized by the excessive secretion of catecholamines, particularly epinephrine and norepinephrine (1). The overproduction of these hormones, synthesized within the chromaffin cells of the adrenal medulla, profoundly raise cardiometabolic risks potentially leading to hypertensive crises, arrhythmias, insulin resistance, and hyperglycemia (2, 3). Although catecholamine release is a hallmark of PPGLs, these tumors may also secrete a variety of other bioactive peptides and hormones, contributing to a more complex clinical presentation. Previous studies have suggested that PPGLs could secrete ectopic substances such as vasoactive intestinal peptide, calcitonin, aldosterone, or parathyroid hormone-related peptide (4). To date, there have been reports of PPGLs that synthesize either ACTH or corticotropin-releasing hormone (CRH), leading to the manifestation of ectopic Cushing syndrome in affected individuals (5-7). Despite these findings, it has not yet been established whether PPGLs could secrete cortisol excess without the overt signs of Cushing syndrome, thereby resulting in mild autonomous cortisol secretion (MACS). A recent pivotal study demonstrated that patients with pheochromocytoma had higher circulating concentrations of cortisol, 11-deoxycortisol, 11-deoxycorticosterone, and corticosterone compared to controls with primary hypertension (8). Nevertheless, the exact mechanisms underlying the dysregulated steroidogenesis in pheochromocytomas, as well as the clinical implications of these findings and their impact on patient prognosis, are not yet elucidated. MACS is frequently associated with metabolic complications such as hypertension, insulin resistance, type 2 diabetes, dyslipidemia, and osteoporosis, which collectively contribute to decreased quality of life and increased mortality (9, 10). In PPGLs, marked by an excess of catecholamines that already raises cardiovascular and metabolic risk, the presence of MACS may further complicate the clinical profile (11).

The present study was designed to investigate the prevalence of MACS in a cohort of patients with PPGLs, using the screening 1-mg dexamethasone suppression test (DST) as the basis for assessment. Furthermore, the clinical presentations, perioperative outcomes, and prognosis of patients with PPGLs were categorized into 2 groups based on the presence or absence of MACS were studied.

## Methods

This retrospective study was conducted at 2 tertiary endocrinology centers, the Department of Endocrinology, Karolinska University Hospital, Stockholm, Sweden, and the Department of Endocrinology, Oncological Endocrinology, Nuclear Medicine and Internal Medicine, University Hospital in Krakow. An analysis was performed on a database containing records of patients with PPGLs who were diagnosed between the years 2005 and 2023.

The inclusion criteria included patients with pheochromocytoma, abdominal and pelvic paragangliomas, without signs of disease dissemination, qualified for adrenalectomy or extra-adrenal surgery as first-line treatment. The diagnosis of PPGL was confirmed for all patients through histopathological examination after surgery. Exclusion criteria included the presence of metastatic PPGLs, other concurrent malignancies, exacerbation of comorbidities, pregnancy, use of selective estrogen receptor modulators, active hepatitis, history of illicit drug use, use of CYP3A4 enzyme inducers or inhibitors, exogenous steroids, and use of antipsychotics or tricyclic antidepressants. As part of the clinical routine, patients of reproductive age taking oral contraceptives had to discontinue these drugs at least 2 months before hormonal testing. In addition, we did not include the group of head and neck paragangliomas because they are considered as a separate group based on their anatomical localization, clinical presentation, molecular pathogenesis, and biological behavior (12).

The diagnosis of PPGL was made clinically on the basis of elevated catecholamines in urine or plasma, as well as imaging, whereas we only included patients with histopathological confirmation of PPGL in the final analyses. Using HPLC 24-hour urinary metanephrines were measured, and liquid chromatography–tandem mass spectrometry plasma metanephrine, normetanephrine, and metoxythyramine were measured. Due to the difference in the determination of metanephrines at the 2 centers (24 hours urinary vs plasma determinations), the number of times above the upper limit of normal (ULN) was calculated for metanephrine concentrations.

In this study, the diagnostic criteria for MACS were applied in accordance with the guidelines of the European Society of Endocrinology (13). Following these recommendations, the DST was performed as a screening test for hypercortisolism. Serum cortisol concentrations were measured post-DST, with concentrations above 1.8 µg/dL (50 nmol/L) considered indicative of MACS if no signs of overt Cushing syndrome were present. The DST was performed twice, and for statistical analysis, the lower of the 2 cortisol values was used. In all patients from the nonsuppressive group, both cortisol concentrations exceeded 1.8 µg/dL, whereas in the suppressive group, both values were below this threshold. ACTH independence was confirmed by measuring serum ACTH concentration using an immunoradiometric method (Brahms, Germany) at 1 center and an automated immunoassay analyzer (Nichols, USA) at the other. At both centers, serum cortisol concentrations were measured by electrochemiluminescence (Roche Diagnostics GmbH, Mannheim, Germany). Serum DHEAS was measured by electrochemiluminescence (Roche Diagnostics GmbH). In selected patients (n = 31) who were diagnosed with adrenal incidentalomas in an inpatient setting, midnight cortisol concentrations were also assessed for overnight measurements, and morning cortisol concentrations were recorded at 6 Am and 8 Am, with the higher concentration used for subsequent statistical analyses.

The data were collected retrospectively using the electronic medical records system at each study center. Patients' demographic characteristics (sex, age, body mass index [BMI]), location and diameter of the tumor, radiological characteristics of the tumor, and comorbidities (diabetes mellitus, prediabetes, arterial hypertension, coronary heart disease, chronic obstructive pulmonary disease, chronic kidney disease) were recorded. All patients who gave informed consent were routinely tested for the presence of variants in at least the *RET, SDHB, SDHD, MAX, menin, NF1, AIP* genes by next-generation sequencing.

According to the European Association for the Study of Diabetes and the World Health Organization, prediabetes was defined by one or more of the following criteria: fasting plasma glucose between 5.6 and 6.9 mmol/L, 2-hour plasma glucose levels during a 75-g oral glucose tolerance test between 7.8 and 11.0 mmol/L, or glycated hemoglobin levels ranging from 5.7% to 6.4% (14). Blood pressure was measured in the outpatient clinic before surgery and at the first follow-up visit after surgery. Postoperative improvement in blood pressure was identified as a decline in systolic and diastolic measurements by a minimum of 10 mm Hg, along with a corresponding decrease in the administration of antihypertensive medications. Intraoperative data included type and duration of anesthesia, blood loss, and type and duration of surgery. Postoperative data covered the incidence of complications, hospital stay duration, and tumor features determined through histopathological examination. Histopathological suspicion of malignant behavior was defined by either a Pheochromocytoma of the Adrenal Gland Scaled Score (PASS) ≥ 4 or the pathologist's evaluation of malignancy in the postoperative report, taking into account features such as capsular and vascular invasion, necrosis, and increased mitotic activity. In addition, the percentage activity of the Ki67 proliferation index was assessed. Patient follow-up data were also collected, including reassessment of the presence of hypertension and diabetes status postsurgery, along with development of metastases, recurrence, or death.

Both the Swedish Ethical Review Authority, Sweden, and the Bioethics Committee of the Jagiellonian University, Krakow, Poland approved the study. Due to the retrospective nature, written informed consent was not considered necessary to participate in this study.

### Statistical Analysis

The cohort was divided into 2 groups: patients with PPGLs exhibiting suppressive cortisol secretion and those with nonsuppressive cortisol secretion based on the DST. Results were reported as mean ± SD or, when applicable, as median and interquartile range (IQR). For the comparison of means between 2 continuous variables with a normal distribution, Student *t*-tests were applied; otherwise, Mann-Whitney *U*-tests were used. Correlations between post-DST cortisol concentrations and patients' clinical data and laboratory results were determined using Spearman rank correlation coefficient. The evaluation of changes in blood pressure and diabetes status before adrenalectomy/surgery and at the first follow-up visit following the procedure was conducted using the McNemar test and the Wilcoxon signed-rank test. The McNemar test was employed for the analysis of paired categorical data, which included variations in diabetes status, while the Wilcoxon signed-rank test was used to determine differences in continuous variables, specifically blood pressure measurements.

Results were considered statistically significant when the *P* value was found to be less than .05. All data were analyzed with StatSoft STATISTICA v.13 and R-Studio (Vienna, Austria; https://www.R-project.org/).

## Results

### Patient Characteristics at Baseline

**Figure 1.**
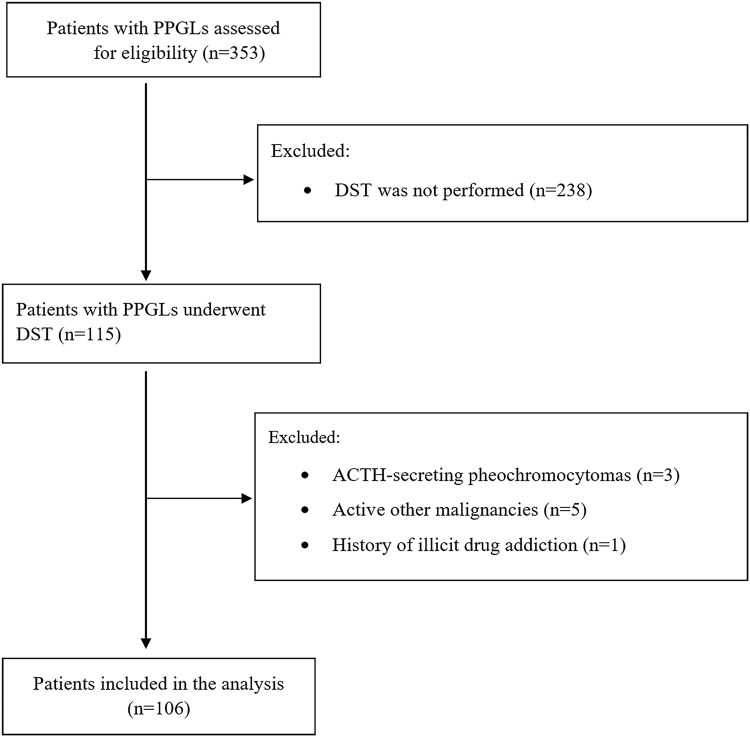
Flowchart of patient inclusion and analysis.

In total, 353 patients with PPGLs were identified, of whom 115 underwent a DST. Three patients were diagnosed with ectopic Cushing syndrome due to ACTH secretion by the pheochromocytoma (0.8% of the total cohort of 353 patients with PPGLs); these cases we described elsewhere (5, 6). In addition, we excluded 5 patients with active other malignancies and 1 patient with a history of illicit drug addiction. Ultimately, the study included 106 patients, of whom 104 were diagnosed with pheochromocytoma and 2 with paragangliomas in the vicinity of the adrenal glands. The process of patient inclusion in the study is illustrated in the flow chart (Fig. 1).

No synchronous bilateral pheochromocytomas were observed in the cohort at diagnosis; however, one patient experienced recurrence in the contralateral adrenal gland 6 years later.

In the cohort of 106 patients, 24.5% patients did not suppress cortisol concentration in the DST. One patient (0.9%) presented with a hypertensive crisis a few hours after dexamethasone administration, requiring 1-day treatment in the coronary care unit. No other precipitating factors could be identified.

To further explore the potential contribution of the contralateral adrenal gland to cortisol secretion, imaging data were reviewed for all patients. Evidence of contralateral adrenal cortical hyperplasia was found in 1 patient from the suppressed group and 1 from the nonsuppressed group (*P* = 1.0). However, no radiological features consistent with an adenoma were identified in the contralateral adrenal gland in any case.

Three-quarters of the cohort presented as an incidentaloma. Nearly all patients (96.0%) received preoperative α-blockade, predominantly with doxazosin (79.0%), administered for a median of 45 days (IQR 27-74). Additionally, 73.3% were concurrently treated with β-blockers. Genetic testing results were available for 70 patients (66.0% of the cohort), and 11 patients (15.7%) were found to carry gene variants related to PPGLs, most commonly in the *NF1* gene (n = 5). All genetic variants were detected in the group that had a normal DST result (Table 1).

**Table 1.** Demographic analysis and tumor characteristics of patients with pheochromocytomas and paragangliomas, with and without cortisol suppression to the 1-mg overnight dexamethasone suppression test

	All (n = 106)	Suppressed (n = 80)	Nonsuppressed (n = 26)	*P* value
Age at diagnosis (years), median (IQR)	59 (49-69)	56 (47-67)	66 (57-75)	.0018
BMI (kg/m^2^), median (IQR)	24.7 (22.2-28.5)	25 (22.5-28.2)	26.4 (21.4-30.1)	.93
Females, n (%)	61 (57.6%)	39 (48.8%)	22 (84.6%)	.0013
Cardiovascular disease, n (%)	23 (23.5%)	13 (17.8%)	10 (40.0%)	.02
Hypertension treated with drugs, n (%)	59 (60.8%)	41 (56.9%)	18 (72.0%)	.18
Diabetes mellitus, n (%)	33 (34.0%)	20 (27.8%)	13 (52.0%)	.03
Chronic obstructive pulmonary disease, n (%)	7 (7.2%)	5 (6.9%)	2 (7.7%)	.86
Chronic kidney disease, n (%)	2 (2.1%)	1 (1.4%)	1 (4.2%)	.41
Tumor in right adrenal gland, n (%)	52 (55.9%)	35 (50.7%)	17 (70.8%)	.08
Size of the tumor (cm), median (IQR)	4.2 (3.0-5.6)	4.0 (3.0-5.0)	5.2 (3.7-7.1)	.03
Unenhanced attenuation value of the tumor in CT (Hounsfield units), median (IQR)	38 (31-43)	36 (30-42)	42 (40-45)	.26
Pathogenic germline variants, n (%)	11 (15.7%)	11 (19.6%)	0 (0%)	.11
Incidentaloma, n (%)	70 (74.5%)	50 (71.4%)	20 (83.3%)	.25
Abdominal paraganglioma, n (%)	2 (1.9%)	0 (0.0%)	2 (7.7%)	.06
Number of typical symptoms of PPGL, median (IQR)	2 (1-4)	2 (1-4)	3 (1-5)	.92
Classic triad, n (%)	12 (12.0%)	9 (12.2%)	3 (12.0%)	.98
Urine metanephrine (µL/24 hours), median (IQR)	1607 (513-3378)	1292 (450-2869)	3819 (1996-7152)	.009
Urine normetanephrine (µL/24 hours), median (IQR)	2182 (920-4572)	1705 (845-3370)	5623 (2182-13474)	.017
Urine metoxythyramine (µL/24 hours), median (IQR)	303 (174-472)	280 (144-441)	399 (296-569)	.15
Plasma metanephrine (nmol/L), median (IQR)	0.70 (0.4-1.7)	0.75 (0.40-1.5)	0.50 (0.40-2.0)	0.86
Plasma normetanephrine (nmol/L), median (IQR)	4.1 (2.1-9.6)	3.5 (2.0-7.9)	8.8 (4.1-16.0)	.04
Plasma metoxythyramine (nmol/L), median (IQR)	0.1 (0.1-0.2)	0.1 (0.1-0.1)	0.15 (0.1-0.2)	.14
Fold increase in metanephrine (plasma/urine) above ULN, median (IQR)	3.3 (1.3-8.6)	3.0 (1.3-7.3)	5.2 (1.3-14.2)	.31
Fold increase in normetanephrine (plasma/urine) above ULN, median (IQR)	5.4 (2.1-14.2)	4.0 (1.9-10.3)	13.6 (3.5-26.7)	.0097
Fold increase in 3-methoxytyramine (plasma/urine) above ULN, median (IQR)	1.0 (0.5-1.9)	1.0 (0.5-1.9)	1.4 (0.9-2.2)	.23
Maximum fold increase of any metanephrine above ULN, median (IQR)	7.7 (3.7-14.7)	6.3 (3.0-11.8)	14.5 (7.0-32.3)	.003
Plasma ACTH (pmol/L), median (IQR)	22.6 (17.0-36.0)	22.8 (16.8-37.4)	17.4 (17.1-34.0)	.65
Plasma DHEAS (µmol/L), median (IQR)	3.0 (1.7-4.7)	3.9 (2.6-5.5)	0.8 (0.6-1.5)	.001
Morning plasma cortisol (µg/dL), median (IQR)	17.6 (15.5-22.0)	17.0 (14.5-19.8)	22.8 (17.9-30.9)	.016
Midnight plasma cortisol (µg/dL), median (IQR)	3.1 (2.0-5.5)	3.0 (2.0-5.2)	4.4 (3.7-6.6)	.32

Abbreviations: IQR, interquartile range; PPGL, pheochromocytoma and paraganglioma; ULN, upper limit of normal.

**Figure 2.**
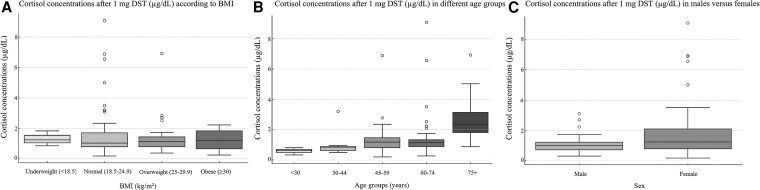
Cortisol concentrations after suppression test with 1 mg dexamethasone (µg/dL) according to (A) body mass index (BMI), (B) age, and (C) sex.

**Figure 3.**
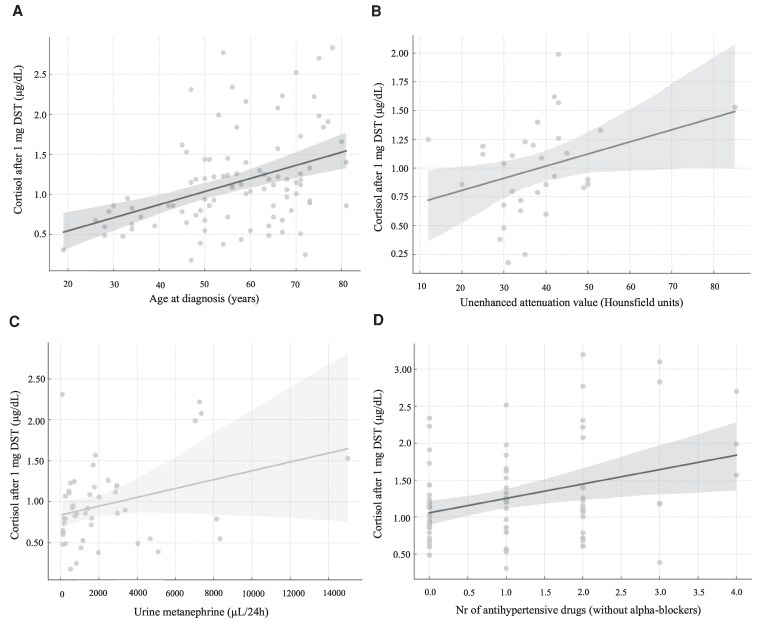
Correlation plots with confidence interval of selected quantitative variables showing significant correlations between cortisol concentrations after the 1-mg dexamethasone suppression test: (A) age at diagnosis (years), (B) unenhanced attenuation value of the tumor in computed tomography (CT) (Hounsfield units), (C) urine metanephrine (µL/24 hours), and (D) number of antihypertensive drugs (in addition to α-blockers).

Patients in the nonsuppressive group were older at diagnosis compared with the suppressive group (66 years [IQR 57-75] vs 56 years [IQR 47-67], *P* = .0018) (Table 1, Fig. 2B). Cortisol concentrations after the DST correlated positively with age at diagnosis (*r* = 0.41; *P* < .0001) (Fig. 3A). Women were more frequently represented in the nonsuppressive group compared with the suppressive group (84.6%, vs 48.8%, *P* = .001) (Fig. 2C). Interestingly, no difference was found between cortisol concentrations after DST and BMI (*P* = .93) (Fig. 2A). Cardiovascular disease and diabetes mellitus were more prevalent in the nonsuppressive group (Table 1). There was no significant difference between the groups in the number of typical symptoms of PPGL (2 [IQR 1-4] vs 3 [IQR 1-5], *P* = .92) or in the frequency of the classic triad (sweating, palpitations, and headache) (12.2% vs 12.0%, *P* = .98).

### Tumor Characteristics and Biochemical Profiles

Patients with nonsuppressive cortisol secretion had larger tumors compared with the suppressive group (5.2 [IQR 3.7-7.1] vs 4.0 [IQR 3.0-5.0] cm, *P* = .03) (Table 1). Cortisol concentrations after DST showed a positive correlation with the unenhanced attenuation value of tumors in computed tomography (*r* = 0.35; *P* = .015) (Fig. 2B).

Urinary metanephrine concentrations were higher in the nonsuppressive group, with a median of 3819 µg/24 hours (IQR 1996-7152) compared to 1292 µg/24 hours (IQR 450-2868) in the suppressive group (*P* = .009) (Table 1, Fig. 2C). Cortisol concentrations after DST correlated positively with urinary metanephrine concentrations (*r* = 0.34; *P* = .009) (Fig. 3C). Similarly, urinary normetanephrine concentrations were higher in the nonsuppressive group compared with the suppressive group (4619 µg/24 hours [IQR 1844-11482] vs 1698 µg/24 hours [IQR 845-4349], *P* = .047) (Table 1). Plasma normetanephrine concentrations were also higher in the nonsuppressive group compared with the suppressive group (8.8 nmol/L [IQR 4.1-16.0] vs 3.5 nmol/L (IQR 2.0-7.9), *P* = .04) (Table 1). However, plasma metanephrine concentrations did not differ between groups. The maximum increase of any metanephrine concentrations above the ULN was higher in the nonsuppressive group compared with the suppressive group (14.5 [IQR 7.0-32.3] vs 6.3 [IQR 3.0-11.8], in *P* = .003) (Table 1).

Cortisol concentrations after DST were also positively correlated with morning cortisol concentrations (*r* = 0.46; *P* = .01) and midnight cortisol concentrations (*r* = 0.40; *P* = .02). There was a nonsignificant lower ACTH concentration in the nonsuppressive compared with the suppressive group (17.4 [IQR 17.1-34.0] vs 22.8 [IQR 16.8-37.4] pmol/L, *P* = .65). However, DHEAS concentrations were significantly lower in the nonsuppressed group compared to the suppressed group (0.8 µmol/L [IQR 0.6-1.5] vs 3.9 µmol/L [IQR 2.6-5.5], *P* = .01).

Morning cortisol was correlated with midnight cortisol concentration (*r* = .4, *P* = .025), cortisol concentration after DST (*r* = 0.35, *P* = .05), and urinary metanephrine (*r* = 0.46, *P* = .015). Midnight cortisol was also correlated with urinary 3-metoxytyramine (*r* = 0.32, *P* = .046). We did not find any correlation between BMI and cortisol measurement (morning, nocturnal, or after DST).

### Surgical and Postoperative Outcomes

The laparoscopic approach was the predominant method for adrenalectomy/surgery in both groups, performed in 89.7% of all patients. In the nonsuppressive group, the frequency of laparoscopic adrenalectomy/surgery was lower compared to the suppressive group (78.3% vs 93.8%, *P* = .036). Conversion to open surgery was noted solely in the nonsuppressive group, affecting 8.3% of the patients (*P* = .02). Additionally, the occurrence of postoperative complications was higher in the nonsuppressive group, with a rate of 29.2%, compared to 10.0% in the suppressive group (*P* = .04) (Table 2). Perioperative complications, blood loss, and hospital stay were all higher in the nonsuppressive group (*P* < .05). No clinical features consistent with severe postoperative adrenal insufficiency were observed in either group. In the nonsuppressed group, 3 patients experienced significant intraoperative hemodynamic instability, characterized by abrupt hypertensive spikes with systolic blood pressure exceeding 200 mm Hg; however, no episodes of hypotension with mean arterial pressure below 60 mm Hg were documented. Importantly, life-threatening complications or perioperative deaths did not occur in our cohort. Further details can be seen in Table 2.

**Table 2.** Surgical outcomes, histopathological findings, and long-term follow-up in patients with pheochromocytomas and paragangliomas, with and without cortisol suppression to the 1-mg overnight dexamethasone suppression test

	All (n = 106)	Suppressed (n = 80)	Nonsuppressed (n = 26)	*P* value
Preoperative doxazosin dose (mg), median (IQR)	16 (8-27)	16 (8-24)	24 (12-32)	.14
Laparoscopic surgery, n (%)	78 (89.7%)	60 (93.7%)	18 (78.3%)	.036
Conversion to open surgery, n (%)	2 (2.3%)	0 (0.0%)	2 (8.3%)	.02
Complications, n (%)*^a^*	14 (14.9%)	7 (10.0%)	7 (29.2%)	.04
Surgery time (min), median (IQR)	90 (70-120)	90 (70-100)	120 (75-130)	.10
Anesthesia time (min), median (IQR)	120 (100-155)	115 (100-130)	150 (100-168)	.33
Blood loss (mL), median (IQR)	55 (30-100)	50 (30-100)	200 (100-350)	.014
Length of hospital stay (days), median (IQR)	3.0 (2.0-4.0)	2.5 (2.0-3.5)	4.0 (2.5-6.0)	.01
Suspected malignancy on histopathology report, n (%)	26 (31.3%)	17 (25.4%)	9 (56.3%)	.03
PASS score, median (IQR)	3 (2-5)	2.5 (2-5)	4 (2-4)	.56
Ki67 (%), median (IQR)	2 (1-3)	2 (1-3)	3 (2-5)	.05
Metastasis and/or recurrence, n (%)	7 (8.0%)	3 (4.7%)	4 (16.7%)	.06
Death, n (%)*^b^*	18 (16.9%)	13 (16.3%)	5 (19.2%)	.73
Other concurrent pathology in the adrenal glands, n (%)	14 (17.7%)	8 (13.3%)	6 (31.6%)	.07
Cortical hyperplasia, n (%)	7 (11.5%)	4 (8.7%)	3 (20.0%)	.23
Adrenal medullary hyperplasia, n (%)	2 (2.4%)	1 (1.7%)	1 (4.2%)	.51
Composite pheochromocytoma, n (%)	4 (6.3%)	2 (4.2%)	2 (12.5%)	.23

Abbreviations: IQR, interquartile range; PASS, Pheochromocytoma of the Adrenal Gland Scaled Score.

^*a*^Perioperative complications included intraoperative hemorrhage (6 patients), hemodynamic instability (3 patients), wound-related complications (2 patients), splenic injury (2 patients), and ileus (1 patient).

^*b*^Causes of death included 9 patients from cardiovascular events (stroke, myocardial infarction, severe heart failure), 1 patient from brain injury in a traffic accident, 2 patients from respiratory failure, 5 patients from disseminated malignancies other than pheochromocytoma (absent at the time of PPGL diagnosis), and 1 patient from disseminated PPGL.

### Blood Pressure and Glycemic Control

Before adrenalectomy/surgery, patients in the nonsuppressive group exhibited higher median systolic blood pressure (160 vs 150 mm Hg, *P* = .05). Furthermore, the nonsuppressive group required more antihypertensive medications, in addition to α-blockers, compared with the suppression group (2 [IQR 2-3] vs 1 [IQR 1-2], *P* = .045). Cortisol concentrations after DST correlated positively with the number of antihypertensive drugs required preoperatively (*r* = 0.22; *P* = .046) (Fig. 3D). Following adrenalectomy/surgery, both groups demonstrated marked improvements in their blood pressure levels and a decrease in the number of antihypertensive agents prescribed (Table 3).

**Table 3.** Blood pressure and glycemic abnormalities in patients with pheochromocytomas and paragangliomas, at diagnosis and at the first follow-up visit after surgery

	All patients (n = 106)			Suppressed (n = 80)			Non-Suppressed (n = 26)			*P* value*^b^*
	At diagnosis	After surgery	*P^a^*	At diagnosis	After surgery	*P^a^*	At diagnosis	After surgery	*P^a^*	
Systolic BP (mm Hg), median (IQR)	149 (135-170)	130 (120-138)	*P* < .0001	145 (130-160)	129 (120-136	*P* < .0001	160 (143-180)	130 (120-143)	.0001	.05/0.58
Diastolic BP (mm Hg), median (IQR)	88 (80-100)	80 (75-85)	*P* < .0001	85 (80-100)	80 (75-85)	*P* < .0001	90 (80-100)	80 (70-80)	.0027	.43/0.22
Number of antihypertensive drugs, median (IQR)	2 (1-3)	0 (0-2)	*P* < .0001	2 (1-3)	0 (0-1)	*P* < .0001	2 (2-3)	0.5 (0-2)	.011	.25/0.80
Number of antihypertensive drugs except α blocker, median (IQR)	1 (0-2)	0 (0-1)	0.005	1 (0-2)	0 (0-1)	0.08	1.5 (1-2)	0 (0-1)	.015	.02/0.90
Improvement BP after surgery (yes vs no)	72 (83.7%)			55 (85.9%)			17 (77.3%)			.34
Diabetes, n (%)	32 (33.3%)	18 (18.8%)	0.01	19 (26.8%)	9 (12.7%)	0.04	13 (50.0%)	8 (33.3%)	0.48	.01/0.035
Diet-controlled diabetes, n (%)	5 (8.3%)	3 (5.9%)	1	3 (6.4%)	1 (2.4%)	1	2 (15.4%)	1 (9.1%)	1	.30/0.30
Diabetes controlled by oral antihyperglycemic agents, n (%)	18 (28.1%)	12 (22.6%)	0.22	12 (23.5%)	3 (7.1%)	0.13	6 (46.2%)	5 (45.5%)	0.48	.11/0.0016
Diabetes controlled by insulin, n (%)	9 (14.8%)	4 (7.5%)	0.25	5 (10.2%)	2 (4.8%)	1	4 (33.3%)	2 (18.2%)	0.48	.04/0.13

Abbreviations: BP, blood pressure; IQR, interquartile range.

^*a*^Comparison of differences before and after adrenalectomy in the same group.

^*b*^Comparison of pre- and postadrenalectomy differences between suppressed and nonsuppressed groups.

The occurrence of prediabetes was comparable between groups (14% vs 8%, *P* = .46). However, diabetes mellitus was more common in the nonsuppressed group both before and after surgery (Table 3). Before adrenalectomy/surgery, insulin dependence was higher in the nonsuppressive group (33.3% vs 10.2%, *P* = .04). After adrenalectomy/surgery, diabetes persisted more in the nonsuppressive group compared with the suppression group (33.3% vs 12.7%, *P* = .04) (Table 3). Among patients with insulin dependency, 25% in the nonsuppressive group and 60% in the suppressive group discontinued insulin therapy after surgery. Moreover, the proportion of patients requiring only oral antihyperglycemic agents postoperatively remained higher in the nonsuppressive group compared to the suppressive group (Table 3).

### Follow-Up

Of the 26 patients who did not exhibit cortisol suppression after DST prior to surgery, only 4 (15%) underwent repeated testing after adrenalectomy; among these, the median cortisol concentration before surgery was 2.8 µg/dL (range 2.0-9.1 µg/dL) (77.3 nmol/L [range 54.9-250.2 nmol/L)], whereas after surgery the median was 0.79 µg/dL (range 0.54-1.44 µg/dL) (21.8 nmol/L [range 14.9-39.7 nmol/L)].

Histopathological evaluation, based on pathological assessment of malignancy, showed a higher suspicion of malignancy in the non-suppressive group compared to the suppressive group (56.3% vs 25.4%, *P* = .03). The Ki-67 proliferation index was higher in the nonsuppressive group compared with the suppressive group (3% [IQR 2-5] vs 2% [IQR 1-3], *P* = .05), whereas PASS score did not differ significantly between groups (Table 2). No substantial differences were identified in the histopathological assessment concerning the prevalence of adrenal cortical hyperplasia, adrenal medullary hyperplasia, or composite pheochromocytoma in the remaining adrenal tissue. Over a median follow-up of 6 years (IQR 4-11), 18 deaths occurred, 17 unrelated to pheochromocytoma, whereas 7 patients experienced recurrence or metastasis. Although there was no difference in the mortality rate between the compared groups (16.3% vs 19.2%, *P* = .72), a trend of more aggressive disease course, expressed as recurrence or metastatic disease, was observed in the nonsuppressive group compared to the suppressive group (16.7% vs 4.7%, *P* = .06).

## Discussion

This study is pioneering in demonstrating that one quarter of patients with PPGLs exhibit features of MACS, which were associated with larger tumor size, higher metanephrine secretion, an increased prevalence of diabetes mellitus, and an increased need for antihypertensive medications. Nonsuppression in the DST was also associated with increased perioperative risks, including higher rates of complications, greater blood loss, and prolonged hospital stays. Notably, the DST results showed a near-significant impact on histopathological malignancy markers (eg, Ki67 index) and the risk of metastases or recurrence, suggesting potential links to tumor aggressiveness that warrant further investigation.

To date, the literature on PPGLs (both pheochromocytomas and paragangliomas have been described) associated with hypercortisolemia has primarily focused on tumors secreting ectopic ACTH and/or CRH, which predominantly present as overt Cushing syndrome (15, 16). In our cohort of all PPGLs only 0.8% produced enough ectopic ACTH and/or CRH to present as overt Cushing syndrome. Reports of MACS in pheochromocytomas have been more scarce (17-19). A breakthrough was made in the study by Constantinescu et al, which demonstrated elevated concentrations of glucocorticosteroid metabolites in patients with pheochromocytomas but not in those with paragangliomas (8). Specifically, the authors identified increased concentrations of cortisol, 11-deoxycortisol, and corticosterone in patients with pheochromocytoma compared with those with primary hypertension. Furthermore, adrenalectomy resulted in significant decline in glucocorticoid concentrations, providing strong evidence that the observed alterations were directly related to the pheochromocytoma, rather than being attributable to age or coexisting comorbidities. Notably, they also identified significant positive correlations between cortisol and both metanephrine and normetanephrine concentrations. These findings are in line with our results, as we also demonstrated significant correlations between morning and post-DST cortisol concentrations and metanephrines. However, in our study, among the 26 patients in the nonsuppressed group, 2 were diagnosed with abdominal paragangliomas with nonsuppressed cortisol concentration post-DST. Although the study by Constantinescu et al shed light on the higher circulating concentrations of glucocorticosteroids in patients with pheochromocytomas, it did not evaluate the frequency of MACS. In addition, it did not address detailed demographic parameters, tumor size, perioperative and histological outcomes, follow-up, or changes in blood pressure and glycemic disturbances. Our study complements and extends these findings by additionally demonstrating the utility of cortisol concentration analysis following the DST, which is now recognized as a dynamic screening tool for hypercortisolemia (13). It is worth emphasizing that despite previously demonstrated ability of pheochromocytomas to produce glucocorticoids, three quarters of patients in our cohort appropriately suppressed cortisol following DST. This finding highlights the absence of MACS in the majority of cases, providing a more comprehensive clinical evaluation of this patient group.

The underlying mechanisms contributing to hypercortisolemia in individuals with PPGLs remain elusive. It is still uncertain whether the PPGLs are the source of cortisol and its metabolites, or if catecholamines affect adrenal cortical steroidogenesis through paracrine pathways (20, 21). This ambiguity has been discussed in the study by Constantinescu et al (8) whose findings suggest that hypercortisolemia observed in patients with chromaffin cell tumors are more likely attributable to locally produced glucocorticoids rather than the effects of circulating catecholamines. In our study, we included a histopathological evaluation of the remaining adrenal tissue, which revealed no solitary adrenocortical adenomas capable of autonomous cortisol production. Furthermore, the frequency of adrenal cortical hyperplasia was comparable between groups that suppressed and did not suppress cortisol after the DST, indicating that hypercortisolemia in pheochromocytomas is unlikely to be associated with concurrent adrenal cortical pathology.

In MACS, ACTH concentrations are typically suppressed as a result of negative feedback from excess cortisol. Suppressed or low-normal morning ACTH concentrations are considered an essential criterion for confirming ACTH-independency in patients with adrenal cortisol-producing adenomas (13). However, in our cohort, ACTH concentrations in the nonsuppressive group tended to remain within the low to normal range rather than being clearly suppressed, as commonly seen in MACS. This observation may indicate a distinct pathophysiological mechanism, in which pheochromocytomas co-secrete cortisol in lesser amounts as a secondary metabolic product of tumor function, in contrast to adrenal tumors primarily dedicated to cortisol production. This could explain the relatively less pronounced suppression of ACTH. Our interpretation aligns with findings from previous studies, including that of Constantinescu et al, which reported intermediate cortisol and steroid metabolite concentrations in patients with pheochromocytoma—higher than in those with primary hypertension but lower than in patients with adrenal Cushing syndrome (8). Moreover, we know that PPGLs may produce ACTH and/or CRH in ectopic Cushing syndrome resulting from PPGLs (15). All cases with ectopic Cushing syndrome due to PPGLs had been excluded in this study; however, the remaining PPGLs may possibly also produce smaller amounts of ACTH. Thus, the low-normal ACTH concentrations may be a combination of both explanations.

Interestingly, it has been proposed that measuring serum DHEAS concentrations may facilitate the identification of patients with MACS (22, 23). Because DHEAS production declines when the hypothalamic–pituitary–adrenal axis (HPA) is chronically suppressed, it may reflect cortisol autonomy even in the absence of overt ACTH suppression. In our cohort, although ACTH concentrations did not significantly differ between groups, DHEAS concentrations were markedly lower in the nonsuppressed group, supporting the hypothesis of underlying HPA axis suppression in these patients.

The DST is generally considered safe and typically does not cause adverse effects (24). However, in our study, 1 patient (0.9%) experienced a hypertensive crisis shortly after dexamethasone administration. Notably, in the literature, only 3 cases of hypertensive crisis have been described following the administration of a high-dose, 2-day dexamethasone suppression test (2 mg every 6 hours), whereas no such events have been reported after a 1-mg overnight DST (25). Thus, this is the first reported case of hypertensive crisis after 1-mg DST.

Our findings revealed a significantly higher prevalence of cortisol nonsuppression among women (84.6% vs 48.8% in suppression group), aligning with previous studies on MACS. Ouyang et al found that women diagnosed with MACS demonstrated elevated cortisol concentrations at both midnight and the following morning after DST, in contrast to males (26). In addition to sex, age also influenced cortisol suppression dynamics. Patients in the nonsuppressive group were older, and cortisol concentrations after DST showed a linear correlation with age. Similar findings were reported by Olsen et al (27) and Singh et al (28), who observed higher cortisol concentrations post-DST in older patients with MACS and adrenal incidentalomas. These findings suggest that both age and sex contribute to differences in cortisol suppression dynamics, likely from the combined effects of hormonal sensitivity and HPA axis regulation. Autonomous cortisol secretion has traditionally been linked to obesity, with studies demonstrating a higher prevalence of overweight and obesity in patients with MACS compared to the general population (29). However, in our PPGL cohort, BMI did not differ between patients exhibiting features of MACS and those without. This observation stands in contrast to previous findings and may be attributable to the counterbalancing metabolic effects of catecholamine excess. Notably, An et al reported an inverse relationship between catecholamine concentrations and BMI in patients with PPGL, alongside a positive correlation between postoperative weight gain and presurgical catecholamine secretion, further supporting the hypothesis that catecholamines may mitigate cortisol-induced weight gain (30).

During recent decades, frequent disturbances in glucose metabolism have been well documented among patients with PPGLs (31-34). Briefly, excess of catecholamines have been shown to disrupt glucose homeostasis through mechanisms such as enhanced glycogenolysis, increased gluconeogenesis, and inhibited insulin secretion, collectively leading to hyperglycemia and insulin resistance (31). Similarly, MACS observed in adrenal incidentalomas has been linked to a higher prevalence of diabetes compared to nonfunctioning adrenal incidentalomas (35, 36). Our analysis demonstrated that individuals with PPGL who did not show cortisol suppression during the DST had a notably higher prevalence of diabetes, both pre- and postsurgery. These findings indicate a cumulative detrimental impact of excess catecholamines and cortisol on glucose metabolism. Notably, patients with features of MACS were more likely to require insulin therapy preoperatively and continued to depend on oral antidiabetic medications postoperatively, underscoring a persistent metabolic burden.

Adrenalectomies/surgeries performed for PPGLs are widely recognized as more technically challenging and associated with a higher risk of perioperative complications compared to those conducted for other indications (37). In our study, we further demonstrated that patients with PPGLs exhibiting features of MACS were associated with an increased likelihood of open surgery, intraoperative conversion, and a higher incidence of perioperative complications. However, tumor size may represent a confounding factor because it has been established as one of the most significant predictors of surgical morbidity in pheochromocytomas (38). Notably, the higher complication rate observed in our study among PPGLs with MACS could be attributed to their significantly larger size compared to those without MACS. On the other hand, previous studies have established that, in addition to tumor size and elevated metanephrine as well as normetanephrine concentrations, a lack of perioperative hydrocortisone treatment is also a risk factor for intraoperative hypertensive crises and perioperative complications (38, 39). In children and adolescents with pheochromocytomas, many use hydrocortisone postoperatively (40). In light of the high prevalence of hypercortisolemia in patients with PPGLs, we advocate for systematic screening for hypercortisolemia in all patients with PPGLs to better assess its clinical implications and optimize perioperative management.

To reduce the risk of confounding in the interpretation of DST results, we applied strict exclusion criteria, excluding patients with metastatic PPGLs, other malignancies, acute exacerbation of chronic diseases, pregnancy, and the use of medications known to interfere with cortisol metabolism. Following their implementation, approximately one quarter of patients in our study met biochemical criteria for autonomous cortisol secretion. This subgroup had larger tumors, a higher prevalence of comorbidities, and greater perioperative risk. Although these findings support a potential role for cortisol excess in shaping the clinical course of PPGL, we are aware that well-established factors such as tumor size and catecholamine excess are also known contributors to comorbidities—particularly glycemic dysregulation—as well as surgical burden. Therefore, the observed differences likely reflect a multifactorial interaction, in which MACS is one of several contributing elements.

The association between hypercortisolemia and the malignant potential of PPGLs remains uncertain. In our cohort, the Ki67 index and recurrence rates in the nonsuppressed group were higher, approaching statistical significance, whereas the PASS score did not differ significantly between groups. It should be noted, however, that although the PASS has traditionally been used to assess the risk of aggressiveness and potential recurrence, its positive predictive value for disease recurrence is only about 30%; this underscores its limitations in reliably predicting long-term outcomes for patients (41). The only certain way to diagnose a malignant PPGL is if metastases are present (42). Determining whether MACS affects the prognosis of patients with PPGL requires further investigation in larger cohorts.

The strengths of this study include its comprehensive analysis of MACS in patients with PPGLs, integrating biochemical, clinical, and histopathological data. Additionally, the study provides novel insights into the interplay between cortisol secretion and catecholamine excess, contributing to a better understanding of their combined impact on metabolic and perioperative outcomes. However, our study is not without limitations. The retrospective design limits the ability to establish causal relationships and introduces potential biases in data collection. Furthermore, PPGLs are rare tumors, which contributes to a limited sample size due to their low prevalence. Our cohort included only 2 patients with paragangliomas, necessitating further investigations in this subgroup of patients with PPGL. Larger studies—preferably multicenter—are needed to confirm our findings and to enable robust multivariable analyses that account for a broader range of potential confounders. Despite some authors suggesting measuring dexamethasone concentrations in blood after the DST to ensure its accuracy, this has not yet become a standard procedure (43). In our study, to improve reliability of the DST, we made efforts to exclude factors such as active malignancies, exacerbation of chronic diseases and medications confounding cortisol metabolism. Another limitation in our study is that postoperative DST was performed in only 4 patients; however, all results fell within the normal range, and clinically, all patients did not exhibit features of hypercortisolism after surgery. In addition, data on perioperative cortisol concentrations and glucocorticoid use in patients with PPGLs were not available, as these parameters were not routinely assessed. Finally, the number of patients with disease recurrence or death may have been insufficient to establish a statistically significant association between mild hypercortisolism and metastatic tumor potential.

In conclusion, one quarter of patients with PPGLs demonstrated MACS. This phenomenon was associated with larger tumor sizes, higher catecholamine concentrations, metabolic disturbances, and increased perioperative risks. These findings underscore the need for routine hypercortisolemia screening in patients with PPGLs and call for further research into the impact of cortisol secretion on tumor behavior, clinical outcomes, and long-term prognosis.

## Data Availability

Restrictions apply to the availability of some or all data generated or analyzed during this study to preserve patient confidentiality or because they were used under license. The corresponding author will on request detail the restrictions and any conditions under which access to some data may be provided.

## Abbreviations

BMI: body mass index
CRH: corticotropin-releasing hormone
DST: dexamethasone suppression test
HPA: hypothalamic–pituitary–adrenal axis
IQR: interquartile range
MACS: mild autonomous cortisol secretion
PASS: Pheochromocytoma of the Adrenal Gland Scaled Score
PPGL: pheochromocytoma and paraganglioma
ULN: upper limit of normal
